# Investigation of Antioxidant and Hepatoprotective Activity of Standardized *Curcuma xanthorrhiza* Rhizome in Carbon Tetrachloride-Induced Hepatic Damaged Rats

**DOI:** 10.1155/2014/353128

**Published:** 2014-07-14

**Authors:** Sutha Devaraj, Sabariah Ismail, Surash Ramanathan, Mun Fei Yam

**Affiliations:** ^1^Centre for Drug Research, Universiti Sains Malaysia, 11800 Minden, Pulau Pinang, Malaysia; ^2^School of Pharmaceutical Sciences, Universiti Sains Malaysia, 11800 Minden, Pulau Pinang, Malaysia

## Abstract

*Curcuma xanthorrhiza* (CX) has been used for centuries in traditional system of medicine to treat several diseases such as hepatitis, liver complaints, and diabetes. It has been consumed as food supplement and “jamu” as a remedy for hepatitis. Hence, CX was further explored for its potential as a functional food for liver related diseases. As such, initiative was taken to evaluate the antioxidant and hepatoprotective potential of CX rhizome. Antioxidant activity of the standardized CX fractions was determined using *in vitro* assays. Hepatoprotective assay was conducted against carbon tetrachloride- (CCl_4_-) induced hepatic damage in rats at doses of 125, 250, and 500 mg/kg of hexane fraction. Highest antioxidant activity was found in hexane fraction. In the case of hepatoprotective activity, CX hexane fraction showed significant improvement in terms of a biochemical liver function, antioxidative liver enzymes, and lipid peroxidation activity. Good recovery was observed in the treated hepatic tissues histologically. Hence, the results concluded that CX hexane fraction possessed prominent hepatoprotective activities which might be due to its *in vitro* antioxidant activity. These findings also support the use of CX as a functional food for hepatitis remedy in traditional medicinal system.

## 1. Introduction

The practice of using natural remedies for the treatment of liver diseases has been historic, starting with the Ayurvedic treatment and extending to the Chinese, European, and other systems of traditional medicines [1]. Currently, medicinal herbs and extracts prepared from the traditional systems have created a major impact in the treatment of liver diseases such as hepatitis, cirrhosis, and loss of appetite [2]. Several herbs are highlighted and have been scientifically investigated for their hepatoprotective effects [3].

CX has been used for centuries in traditional system of medicine to treat several diseases. In folk medicine, CX is reported to be useful for hepatitis, liver complaints, diabetes, rheumatism, cancer, hypertension, and heart disorders. CX has also shown diuretic, anticancer, anti-inflammatory, antioxidant, antihypertensive, antirheumatic, antihepatotoxic, antidysmenorrheal, antispasmodic, antileucorrhoeal, antibacterial, and antifungal effects. Traditionally, this plant which is available as a herbal drink prevents blood clots and increases the immune system [4]. CX is very often utilized as an ingredient in “jamus” recipe which is a typical Indonesian kind of elixir or liquid remedy [5]. There are several claims that CX has been used for its hepatoprotective purposes in folk medicine. This was supported by lowering of the serum enzyme levels such as alanine aminotransferases (ALT), aspartate aminotransferases (AST), and y-glutamate transferases in cisplastin-induced hepatotoxicity in rats given the CX extract [6]. In addition, the hepatoprotective activity of aqueous extract of CX against *β*-D-galactosamine-induced liver damage and alcohol has been reported by Lin et al. [7] and Yasni et al. [8], respectively. However, there are lacking evidences in relating the antioxidant competence with hepatoprotective properties of this plant. In view of this, the present study was aimed at evaluating the hepatoprotective and antioxidant activity of CX rhizome on rat liver damage by carbon tetrachloride (CCl_4_) which has the potential to be developed as nutraceutical liver supplement for the well being of consumers.

## 2. Materials and Methods

### 2.1. Plant Materials

CX plants were obtained from Johor Plantation, Malaysia. A voucher specimen (11022) was authenticated and deposited at the Herbarium Unit of the School of Biological Sciences, Universiti Sains Malaysia.

### 2.2. Preparation of the Plant Material

The rhizome portion of CX was purchased in powder form from Chemical Engineering Pilot Plant (CEPP), UTM, Skudai, Johor, Malaysia. The coarsely powdered material (800 g) was macerated with 8 L of absolute ethanol for 72 hours with occasional shaking. The maceration was repeated thrice. The extract was filtered and concentrated at reduced pressure on rotary evaporator resulting in dark yellow colored mass (yield 5.2%) [9].

### 2.3. Fractionation of CX Ethanolic Extract

50 g of CX ethanolic extract was suspended in water and partitioned with hexane (15 times), ethyl acetate (EtOAc) (10 times), *n*-BuOH (*n*-butanol), and aqueous fraction, respectively, using a separating funnel. Each fraction was dried under reduced pressure at 40°C to yield 4 different fractions of CX ethanolic extract. Fractionation of CX ethanolic extract was carried out using different solvents, namely, hexane, ethyl acetate, *n*-butanol, and water, respectively. Hexane fraction showed the highest yield of 59.5%, followed by ethyl acetate (39.56%) and water (0.94%). *N*-Butanol fraction did not produce any yield.

### 2.4. Quality Control and Chemical Characterization of CX Fractions Using Gas Chromatography-Mass Spectrometry

Chemical characterization and quality control of the CX fractions (hexane, ethyl acetate, and water) were performed based on the validated method described by [9].

### 2.5. Antioxidant Assay of Standardized CX Fractions

The antioxidant activity of standardized CX fractions was determined using four different assays based on the reported protocols, namely, total phenolics content [10]; total flavonoids content [11]; ferric-reducing antioxidant power assay (FRAP) [12]; di(phenyl)-(2,4,6-trinitrophenyl)iminoazanium (DPPH) scavenging assay [13]; and 2,2′-azinobis-(3-ethylbenzothiazoline-6-sulfonic acid) (ABTS) assay [14]. For each assay, CX fractions and standards were diluted (1 mg/mL) in methanol. Samples were analyzed in triplicate.

### 2.6. Hepatoprotective Assay of Standardized CX Hexane Fraction

#### 2.6.1. Preparation of Stock Solution

1% of sodium carboxymethylcellulose (CMC) stock solution was prepared in distilled water to be used as vehicle. CCl_4_ stock solution was prepared by 1 : 1 dilution using olive oil [7].

#### 2.6.2. Preparation of Sample and Standard Drug

Standardized CX hexane fraction was obtained from liquid-liquid extraction. This hexane fraction was then filtered and evaporated to dryness. The standardized CX hexane fraction at different doses (125, 250, and 500 mg/kg) and standard hepatoprotective drug, silymarin (100 mg/kg), were dissolved in 1% CMC to be administered orally to the rats, respectively. All the samples were freshly prepared on the day of experiment.

#### 2.6.3. Experimental Animals

Male Sprague Dawley rats (150–200 g) were obtained from the Animal House, Universiti Sains Malaysia. The animals were acclimatized to laboratory conditions for seven days prior to the experiments. Six rats were housed per polycarbonate cage, with free access to food (normal laboratory chow, Gold Coin) and tap water* ad libitum*. The animals were maintained at room temperature under a light/dark cycle of 12 h. Experimental protocols and procedures employed in this study were approved by the Animal Ethics Committee of the School of Pharmaceutical Sciences, Universiti Sains Malaysia, with the reference number USM/PPSF/50(054) Jld 2.

#### 2.6.4. Experimental Design

Experimental protocol was based on previously reported studies with slight modifications [15]. Animals were divided into six groups, each group containing six rats.

- Group 1 served as normal control and received only the vehicle (1% CMC) (1 mL/kg/day) orally for seven consecutive days.
- Group 2 received a single dose of CCl_4_ (1 mL/kg) at day 7.
- Group 3 was pretreated with standard hepatoprotective drug, silymarin 100 mg/kg, orally for seven consecutive days followed by single oral dose of CCl_4_ (1 mL/kg) orally at day 7.
- Groups 4, 5, and 6 were administered with standardized CX hexane fraction (125, 250, and 500 mg/kg body weight) orally, respectively, for seven consecutive days followed by a single dose of CCl_4_ (1 mL/kg) orally at day 7.

#### 2.6.5. Biochemical Parameters Examination

Animals were sacrificed 24 h after the last treatment with at least 16 h of overnight fasting. About 1.5 mL of blood was collected via cardiac puncture using a needle (size 0.50 × 16 mm, Terumo) [16]. Blood samples obtained from the rats were allowed to clot at room temperature for 60 min. Then, the clotted blood samples were centrifuged at 3,000 rpm at room temperature for 15 min to obtain the blood serum. The serum was subjected to biochemical tests such as alanine aminotransferase (ALT), aspartate aminotransferase (AST), alkaline phosphatase (ALP), triglyceride, and total protein (TP) using biochemistry analyzer at Lam Wah Ee Hospital, Penang, Malaysia.

#### 2.6.6. Histopathological Studies

After the animals were sacrificed, postmortem examination was performed according to Tsung et al. [17]. All the organs were sliced into small pieces and preserved in 4% formalin before further treatment. Then the organs were dehydrated using solvents followed by waxing and clearing process. After that, the tissues were dipped into paraffin, cut into 4-5 *μ*m thick sections, and subsequently fixed onto the slides. Finally, samples were stained using hematoxylin-eosin (H & E) and assessed for any tissue damage under photomicroscope.

#### 2.6.7. Antioxidative Enzymes Analysis

Liver homogenates of 10% (w/v) were prepared in an ice cold (4°C) buffer of 0.1 M Tris (hydroxymethyl) aminomethane-HCl (TRIS-HCl), pH 7.4. Using a homogenizer fitted with a teflon pestle the sample was homogenized at 1000 rpm for 2 min. The homogenates were then centrifuged at 1000 rpm at 4°C for 10 min to remove nuclei and debris [18]. The supernatants were stored at −80°C until the time of biochemical analyses including the total protein (TP), superoxide dismutase (SOD), catalase (CAT), glutathione peroxidase (GPx), glutathione reductase (GR), lipid peroxidation, and malondialdeyde (MDA) tests.

#### 2.6.8. Statistical Analysis

The data are expressed as mean ± S.E.M. To determine whether there is any statistical difference among the various groups of subject, one-way ANOVA analysis was carried out followed by Tukey's multiple comparison test using SPSS Version 12 software. A value of *P* < 0.05 was considered as statistically significant.

## 3. Results 

### 3.1. Standardization of CX Fractions

**Figure 1:**
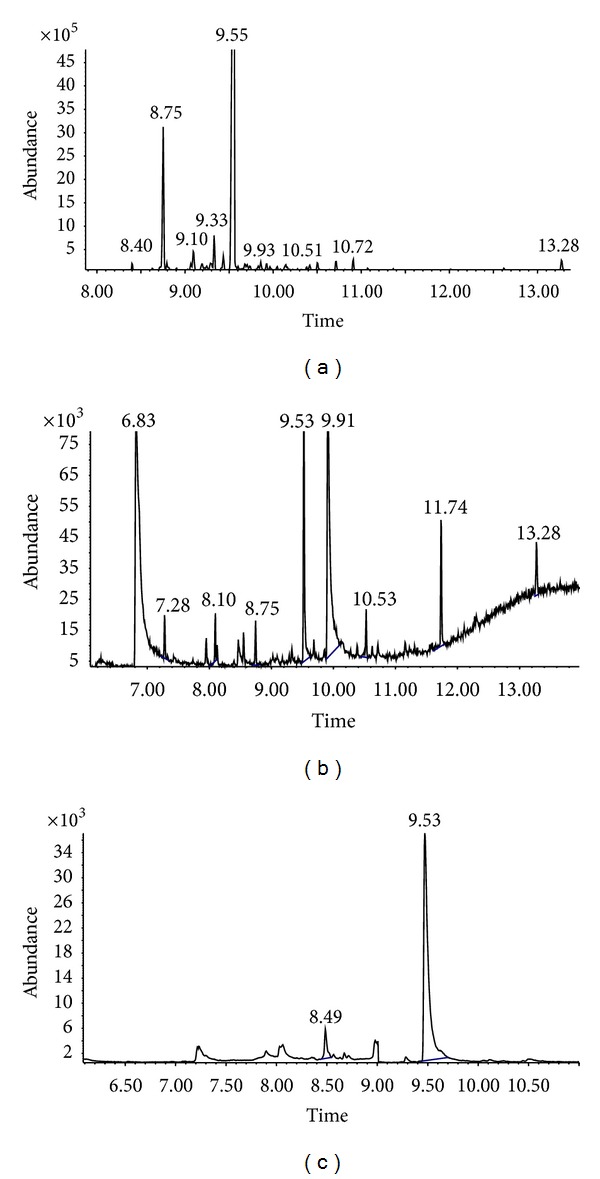
GC-MS fingerprints (nonpolar column) of CX and its fractions. (a) Hexane fraction; (b) ethyl acetate fraction; and (c) water fraction.

Figure 1 shows the GC-MS chromatogram of hexane fraction, ethyl acetate fraction, and water fraction. One prominent peak at approximately 9.55 min was observed for all the fractions injected. This peak is attributed to xanthorrhizol, where the identity was confirmed by matching the retention time and mass spectra provided by MS library and purchased marker standard (xanthorrhizol). As for the standardization, the quantitation of xanthorrhizol was based on the peak area calculated from the calibration curve equation (*y* = 338378*x* − 66068, *r* ^2^ = 0.998) [9]. Xanthorrhizol amount was the highest in hexane fraction (1.8%) followed by ethyl acetate fraction (0.046%) and the least in water fraction (0.03%).

### 3.2. Antioxidant Activity of Standardized CX Fractions

In general, the hexane fraction of CX was found to have higher phenolics and flavonoids content compared to the ethyl acetate and water fractions (Table 1). Ferric reduction activity of hexane fraction is markedly (*P* < 0.05) higher than that of the ethyl acetate fraction and water fractions, respectively, as shown in Table 2. The potential of CX fractions to scavenge free radicals was assessed by their ability to quench DPPH. The IC_50_ of the extracts and standards was arranged in the order of increasing magnitude; water < ethyl acetate < hexane < ascorbic acid < morin < rutin < quercetin. As such, hexane fraction of CX displayed the highest antioxidant properties with the IC_50_ value of 0.035 ± 0.008 mg/mL (*P* < 0.05) compared to the other fractions (Table 2). As for ABTS assay, the results were in similar trend compared to those obtained in the DPPH assay. From the inhibitory concentration (IC_50_) of the fractions as summarized in Table 2, it was seen that the hexane fraction had the highest ABTS^+^ radical scavenging activity as shown by the lowest value of IC_50_.

### 3.3. Hepatoprotective Activity of Standardized CX Hexane Fraction

#### 3.3.1. Effects of Standardized CX Hexane Fraction on Biochemical Enzymes Analysis in CCl_4_-Induced Hepatotoxicity Model

**Figure 2:**
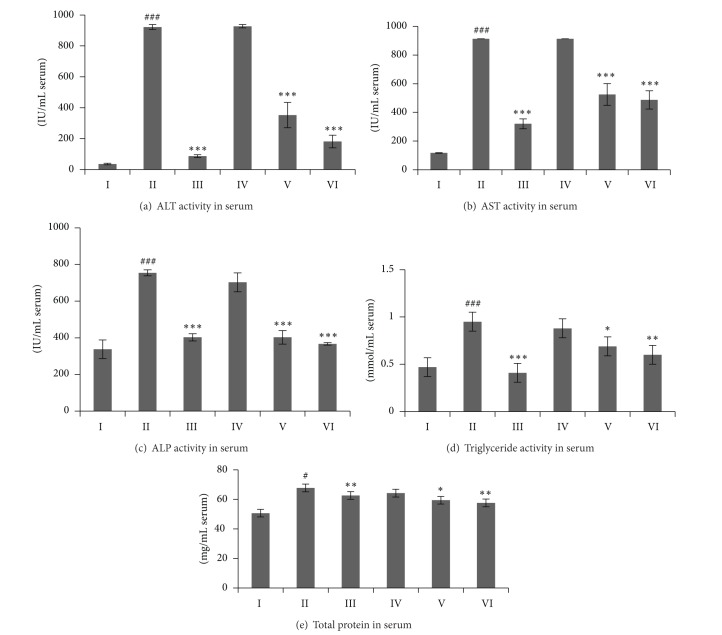
Effects of standardized CXRH on biochemical serum enzymes tests in CCl_4_-induced hepatotoxicity model. Values were expressed as mean ± S.E.M for six animals per group. ^#^ *P* < 0.05, ^##^ *P* < 0.01, and ^###^ *P* < 0.001 were significantly different compared to the control group. *P < 0.05, **P < 0.01, and ***P < 0.001 were significantly different compared to the CCl_4_-treated group. Groups: I (control); II (CCl_4_-treated group); III (100 mg/kg silymarin treated group); IV (125 mg/kg CX treated group); V (250 mg/kg CX treated group); and VI (500 mg/kg CX treated group).

The effect of standardized CX hexane fraction on biochemical and enzymes analysis in CCl_4_-induced hepatotoxicity model in rats is shown in Figure 2. ALT, AST, ALP, triglyceride, and TP were increased significantly by CCl_4_ administration in all treated groups compared to the normal group. However, treatment with standardized CX hexane fraction at 500 mg/kg for 7 days consecutively decreased the ALT, AST, ALP, triglyceride, and TP levels by 40–80%, respectively.

#### 3.3.2. Effects of Standardized CX Hexane Fraction on Histopathology Studies in CCl_4_-Induced Hepatotoxicity in Rats

**Figure 3:**
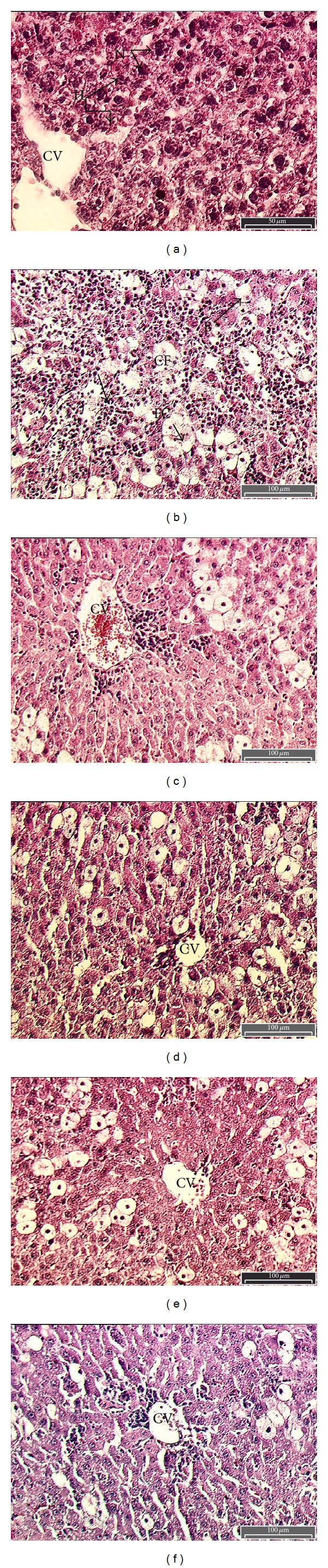
Photomicrograph of a section of liver of rat obtained from different treatment groups. (a) Contol; (b) CCl_4_ control; (c) silymarin (100 mg/kg); (d) CX hexane extract (125 mg/kg); (e) CX hexane extract (250 mg/kg); and (f) CX hexane extract (500 mg/kg). Central vein (CV); round nucleus (N); reticular fibers (RF); massive necrosis (N); hepatocytes ballooning (B); clear cell foci (CF); and fatty change (FC) (H and E; ×40).

In control group, normal hepatic cells are characterized by well defined cell linings, prominent nucleus, and prominient central vein surrounded by reticular fibers (Figure 3(a)). On the contrary, massive necrosis formation, hepatocytes ballooning, distortion of hepatocytes, shrinkage of nucleus, clear cell foci formation, loss of cellular boundaries, and reticular fibers were observed in CCl_4_-intoxicated rats liver section thus indicative of extensive liver injuries (Figure 3(b)). Pretreatment of standardized CX hexane fraction at 125 mg/kg partly prevented hepatoprotective activity. The histopathological changes such as necrosis, ballooning, clear cell foci formation, and structural loss of hepatic lobules were moderate in 250 mg/kg hexane fraction treated groups. However, the histological architecture of liver sections of the rats treated with standardized CX hexane fraction at 500 mg/kg showed almost normal lobular pattern with a mild degree of necrosis, ballooning, clear cell foci, and visible reticular fibers around central vein almost comparable to the control and silymarin treated group (Figures 3(c), 3(d), 3(e), and 3(f)).

#### 3.3.3. Effects of Standardized CX Hexane Fraction on Antioxidative Enzymes in CCl_4_-Induced Hepatotoxicity in Rats

**Figure 4:**
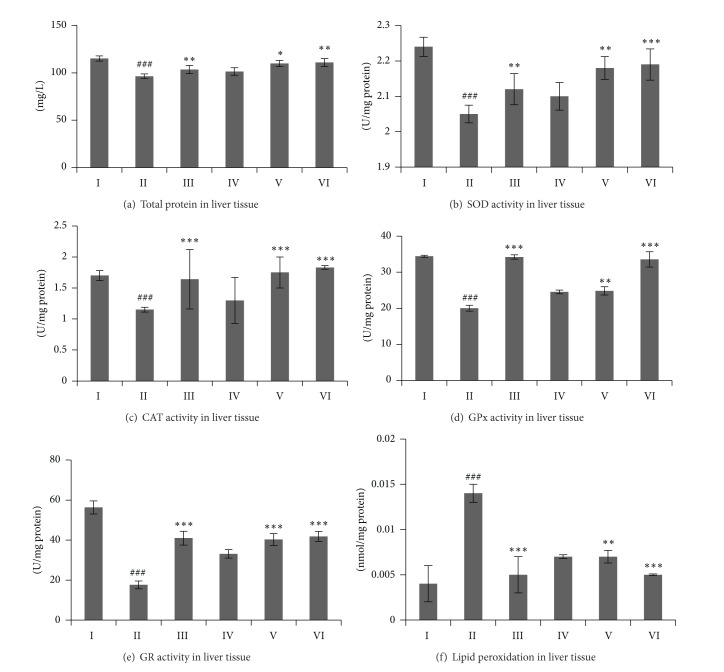
Effects of standardized CXRH on antioxidative enzymes tests in CCl_4_-induced hepatotoxicity model. Values were expressed as mean ± S.E.M for six animals per group. ^#^ *P* < 0.05, ^##^ *P* < 0.01, and ^###^ *P* < 0.001 were significantly different compared to the control group. *P < 0.05, **P < 0.01, and ***P < 0.001 were significantly different compared to the CCl_4_-treated group. Groups: I (control); II (CCl_4_-treated group); III (100 mg/kg silymarin treated group); IV (125 mg/kg CX treated group); V (250 mg/kg CX treated group); and VI (500 mg/kg CX treated group).

The effect of single oral dose of CCl_4_ in rats exhibited significant reduction in TP level and SOD, CAT, GPx, and GR enzyme activities in comparison to the normal (control) group as shown in Figure 4. However, all these enzyme levels were significantly increased by 90% on average at 500 mg/kg of standardized CX hexane fraction. In addition, as illustrated in Figure 4(f), marked inhibition in lipid peroxide (MDA) was observed after pretreatment with standardized CX hexane fraction, and this decrease was recorded at 35.71% in 500 mg/kg treated groups, respectively. The effects of 500 mg/kg of standardized CXRH on the respective enzyme activities were comparable with the silymarin treated group.

## 4. Discussion

Production of active radicals, including oxygen free radicals and nonoxygen free radicals, is a well known phenomenon in normal metabolism process. However, excessive free radicals known as reactive oxygen species (ROS) are potential toxic hazards to various biological molecules through lipid peroxidation [19], DNA damage [20], and inhibition of protein synthesis [20]. Such damage results in various diseases such as cancer, hepatic injury, arteriosclerosis, and reperfusion injury. In this study, CCl_4_ was used as the hepatotoxic agent to examine the hepatoprotective properties of CX. The basis of its hepatotoxicity lies in its biotransformation by the cytochrome P450 system to two free radicals, trichloromethyl free radical and trichloromethylperoxy free radical [21]. Since free radicals play such an important role in CCl_4_-induced hepatotoxicity, plant antioxidants are promising hepatoprotective agents against liver lesion induced by such compounds [18]. In this study, CCl_4_ treatments could modify liver function, since the activities of ALT, AST, ALP, triglyceride, and TP levels were significantly higher compared to the control group. However, the results showed that pretreatment of rats with CX hexane fraction effectively protected the animals against CCl_4_-induced hepatic destruction, as evidenced by decreased serum AST, ALT, and ALP, triglycerides, and TP activities. These biochemical findings were further substantiated by histopathological studies which caused a subsequent recovery of liver cells towards normalization. Antioxidant enzymes (SOD, GPx, GR, and catalase (CAT)) represent protection against oxidative tissue damage [22]. CCl_4_ caused a decrease in GPx, SOD, GR, CAT, and TP activities and increased MDA levels in the liver over those of the control group, implying increased oxidative damage to the liver. However, hexane fraction pretreatment (250 mg/kg and 500 mg/kg) returned the increased MDA and decreased antioxidant enzymes levels back to their control levels, indicating that CX extract may prevent the peroxidation of lipids by CCl_4_. Following the inhibition of lipid peroxidation or upregulation of the antioxidant enzymes activity, NF-E2-related factor 2 (Nrf2) plays an important role which is highly expressed in detoxification organs, such as liver and kidney. Under normal conditions, Nrf2 is located in the cytoplasm where it forms an inactive complex with its repressor Kelch-like ECH2-associated protein. Upon cell stimulation, Nrf2 dissociates from Keap 1, translocates into the nucleus where it binds to ARE, promotes the expression of Nrf2 target genes, and then increases the effect of antioxidative enzymes, such as CAT, SOD, and GSH-Px [23]. Therefore, upregulation of Nrf2 in nuclear can result in a reduction in the level of the reactive metabolites and, correspondingly, less tissue injury. However, this requires further investigation on the cellular mechanism involved in* in vivo* regulation of antioxidant enzymes [23]. The possibility of the mechanism of hepatoprotection of CX may be due to its antioxidant action either by scavenging the reactive oxygen molecules or by chemically reducing oxidized compounds as shown in the findings. The high content of major compound, xanthorrhizol, in the CX hexane fraction could be responsible for the antioxidant and hepatoprotective activity but this warrants further investigation to support such a claim. However, the preventive effect of xanthorrhizol on cisplatin-induced hepatotoxicity has been reported in mice in which they attributed the hepatoprotective activity to regulation of gene transcription [6].

## 5. Conclusion

In conclusion, this study heavily supports the use of CX as source of natural antioxidants and as a possible food supplement for a healthy liver, in view of the fact that this plant encompasses hepatoprotective activity. As such, comprehensive investigation on phytochemical studies of CX is a requisite as plant constituents have significant contribution to the overall bioactivity. Further, the promising results from the activity bring supportive data for detailed mechanism studies as well.

## Figures and Tables

**Table 1:** Total phenolic and total flavonoid content of standardized CX and its fractions.

Samples	Total phenolic content as gallic acid equivalents (GAE) (mg/g extract)	Total flavonoid content as catechin equivalents (CE) (mg/100 g extract)
Hexane	61.00 ± 0.030^a^	92.80 ± 0.009^a^
Ethyl acetate	39.00 ± 0.014^b^	68.60 ± 0.100^b^
Water	0.752 ± 0.015^c^	10.60 ± 0.106^c^

Values are presented in mean ± S.E.M (*n* = 3). Different letters indicate significant difference at *P* < 0.05 for hexane and water fractions.

**Table 2:** Antioxidant properties of standardized CX, its fractions, and standard antioxidants.

Samples	FRAP (*μ*M Fe(II)/g)	IC_50 _value (mg/mL)	
		DPPH free radical scavenging activity	ABTS assay
Hexane	2741.500 ± 21.00^a^	0.035 ± 0.008^a^	0.005 ± 0.001^a^
Ethyl acetate	2529.000 ± 9.000^b^	0.279 ± 0.042^b^	0.020 ± 0.001^b^
Water	2061.500 ± 3.000^c^	1.942 ± 0.123^c^	0.928 ± 0.314^c^

Ascorbic acid	2826.500 ± 15.00^a^	0.025 ± 0.001^a^	0.040 ± 0.018^b^
Quercetin	2366.500 ± 13.00^b^	0.013 ± 0.001^a^	—
Morin	—	0.022 ± 0.009^a^	—
Rutin	—	0.018 ± 0.004^a^	—

Values are presented in mean ± S.E.M (*n* = 3). Different letters indicate significant difference at *P* < 0.05 for different extracts and fractions.
